# Effect of Long-Term Storage on Mycobiota of Barley Grain and Malt

**DOI:** 10.3390/plants10081655

**Published:** 2021-08-11

**Authors:** Soňa Felšöciová, Przemysław Łukasz Kowalczewski, Tomáš Krajčovič, Štefan Dráb, Miroslava Kačániová

**Affiliations:** 1 Department of Microbiology, Faculty of Biotechnology and Food Sciences, Slovak University of Agriculture, Tr. A. Hlinku 2, 949-76 Nitra, Slovakia; sona.felsociova@uniag.sk; 2 Department of Food Technology of Plant Origin, Poznań University of Life Sciences, 31 Wojska Polskiego St., 60-624 Poznań, Poland; 3 Heineken Slovakia Sladovne, a.s., Novozámocká 232/2, 947-01 Hurbanovo, Slovakia; tomas.krajcovic@heineken.com (T.K.); stefan.drab@heineken.com (Š.D.); 4 Department of Fruit Science, Viticulture and Enology, Faculty of Horticulture and Landscape Engineering, Slovak University of Agriculture, Tr. A. Hlinku 2, 94-976 Nitra, Slovakia; 5 Department of Bioenergetics, Food Analysis and Microbiology, Institute of Food Technology and Nutrition, University of Rzeszow, Cwiklinskiej 1, 35-601 Rzeszow, Poland

**Keywords:** malting barley, malt, filamentous fungi, yeast, mycotoxins

## Abstract

Contamination of malting barley grain and malt with micromycetes sampled at various periods post-harvest (3rd, 6th, and 9th month of storage) and types of storage (storage silo and floor warehouse) was investigated. Each of these barley grain samples was malted. This article reports on the changes in the fungal microbiome composition and their overall count in barley grain and malt. From the surface-disinfected barley grain samples collected immediately after harvest, there were eight genera isolated, with a predominance of *Alternaria*. A small increase of isolated microfungi was detected in barley stored in silo for 3 and 6 months (from 142 isolates to 149) and decreased below the number of isolates in barley before storage (133 isolates). Fungal count during storage gradually decreased up to 9 month in barley stored in floor warehouse (from 142 isolates to 84). The initial total count of microscopic fungi in malt before storage was the highest (112 isolates) with 7 genera detected, compared to malts prepared from barley stored for longer time (54 isolates, 7 genera, 9th month of storage). *Alternaria* was the most abundant and frequent genus. Quantitative representation of the filamentous microscopic fungi was lower compared to yeasts especially in barley and malt prepared from barley stored at third month of storage in both type of storage. Yeasts were identified from all grain samples and malt samples with mass spectrometry. Most attention was given to the widely distributed fungus *Penicillium*, 79% of strains produced at least one mycotoxin detected under in vitro assays using the TLC method (97% of them produced griseofulvin, 94% CPA, 79% patulin, 14% roquefortin C, and penitrem A was produced by two screening strains under laboratory conditions). It is therefore important to monitor the microflora throughout the production cycle of “barley to beer”.

## 1. Introduction

Many factors can influence the microbiological contamination of cereal grains during cultivation, harvesting, drying, and their further storage [1]. Microorganisms can come from several sources, including air, dust, water, soil, insects, birds, and rodent feces. Contamination from harvesting equipment or grain processing and the conditions of storage are also important. The weather conditions during plant growth (such as, drought, rainfall, temperature, and sunlight) and even the growing region also influence the type of microbial contamination of the grain [2,3].

In general, the microbial community found on or in the barley seeds may contain numerous species from five groups—viruses, bacteria, fungi, micromycetes, and protozoa. Bacteria together with fungal organisms are the groups with the greatest perceived influence on the properties of barley grain. This is because they occur regularly in higher numbers and many of them are physiologically able to use the grain as nutrient source. The community of fungal species on or in barley seeds and malt grain is subject to change through the production process, starting from the developing ear and grain, through harvested grain, and ending with the kilned malt [4]. The presence of fungal pathogens and mycotoxins in grain is natural in character and the prevention of such occurrences is difficult even if good agricultural practices are followed [5]. The filamentous fungi occurring in grain can be divided into field fungi that occur on the grain until harvest, and storage fungi occurring after harvesting. Field fungi generally do not grow below the water activity (a_w_) of 0.90, corresponding to 20 to 25% moisture, whereas storage fungi are often xerophilic and can grow at a_w_ of 0.80 (18% moisture) or even 0.68 (14% moisture) [6,7]. Typical field fungi such as *Alternaria* spp., *Aureobasidium* spp., *Cladosporium* spp., *Curvularia* spp., *Drechslera* spp., *Epicoccum nigrum*, *Fusarium* spp., *Microdochium* spp., *Nigrospora* spp., *Septoria* spp., and *Trichoderma* spp. dominate the fungal community because they are able to use the developing grain as substrate without damaging or killing the embryo. However, many other fungal species representing all major taxonomic groups can be found upon plating of whole barley grains or dilutions of barley malt. Under storage, these fungi can survive for extended periods of time and will germinate and grow as the water content of barley exceeds 14 to 15% and CO_2_ accumulates at elevated temperatures [8]. The spectrum of fungal species identified from a given barley sample may vary greatly in time, especially during the storage period. This is because many species in the genera *Aspergillus* and *Penicillium*, but also typical xerophiles such as *Eurotium* spp. or *Wallemia sebi*, only start to develop and multiply after harvest, when the water activity of grains decreases to low values during drying and storage [4].

Although the conditions in grain storage facilities are not conducive to the development of field fungi, these fungi can infect the grain while still in the field and continue to grow during storage [9]. Most species of *Penicillium* are xerophiles and are classified as storage fungi, but they can also infect the grain before harvesting. For example, *P. oxalicum* can infect damaged maize grains before harvesting [10]. According to Hill and Lacey [11], up to 85% of barley grains at harvest can be colonized by yeasts, including pink yeasts such as *Sporobolomyces* and *Rhodotorula* [12]. Other yeast species found on barley include *Hansenula, Torulopsis, Candida*, and *Saccharomyces* [12]. Harvesting insufficiently dry grain necessitates additional drying. When conducted incorrectly, that may cause spoilage of cereals, as it promotes the growth of microorganisms and may result in increased levels of mycotoxins [1]. If drying is delayed and the moisture content of the harvested grain is suitable, growth of the field fungi, e.g., *Fusarium* spp., may occur [12]. The mycobiota of stored grains predominantly consist of the ubiquitous mold genera *Aspergillus* (more common at ambient temperatures 20 to 25 °C)*, Alternaria, Cladosporium, Fusarium, Mucor, Rhizopus,* and *Penicillium* (dominant at cooler temperatures) [1]. After harvesting, barley can be stored for some time before malting, but during storage time microbes may still develop. Storage conditions must be carefully monitored to ensure adequate low humidity and low temperature to minimize the microbial growth [13,14] that can have a very negative impact on the quality of beer [15]. Integrated management of mold spoilage risks in stored grain is based on five principles: prevention of mold development by keeping grain moisture below the critical limit of fungal growth; accurate monitoring of increase a_w_ and temperature changes during the storage period, associated with the monitoring of early indicators of respiration activity of storage fungi; reduction of moistening trends in the bulk grain by physical intervention means; use of physical treatments (ozone, grain peeling, or abrasion) to limit mycotoxin contamination transfer to processed cereal products; and possible use of bio-competitive strains of fungi or bacteria to prevent the development of mycotoxigenic fungi in grain bulks [16].

Malting is a process in which a complex ecosystem evolves due to the prevailing moisture and temperature conditions, thus allowing the contaminating microorganisms to develop and to negatively influence the quality of malt [7,17,18]. During malting, the fungal community and other microorganisms growing compete with the grain metabolism for oxygen and may therefore considerably reduce grain germination [6]. Significant increases in levels of several mycotoxins can occur in addition to fungal growth during that same process [19,20]. Several *Fusarium* species were demonstrated to proliferate from steeping through germination until early stages of kilning [19,20] and also certain heat-resistant fungi, such as *Rhizopus* and *Mucor*, and some *Ascomycetes* continue to grow during the early hours of kilning [21].

The proliferation of these fungi is stimulated with higher grains moisture content, higher temperature during storage, long storage period, and intensive infection by fungi before mites. Therefore, it is important to identify the species of fungi in stored barley grains with special emphasis on mycotoxigenic species, which pose a potential risk to human and animal health. This work is a report on the evaluation of the mycobiota of barley and malt grains during storage under various conditions.

## 2. Results

### 2.1. Endogenous Mycobiota of Barley

The filamentous fungi identified from surface disinfected barley grain before and during storage in silo are listed in Table 1, whereas the isolation frequency and relative density are presented in Table 2. The results indicate that mycological colonization of barley grain before and during storage may be diverse and a subject to change during storage. From the barley samples taken immediately after harvest, there were isolated 8 genera and *Mycelia sterilia* (isolates without sporulation) of specific microscopic fungi belonging to the field ecosystem. Of all the 142 strains found, species of *Alternaria* (58% RD) and *Fusarium* (13% RD) predominated, particularly in the Laudis variety. Higher genera representation (7) was obtained from the Kangoo variety, but the highest number of isolates was from the Laudis variety. Isolates of genera *Alternaria, Arthrinium*, and *Epicoccum* were found in all tested samples.

Compared to the baseline of freshly harvested grain, a small increase of detected filamentous fungi was observed in barley stored for three or six months, except for the Laudis variety with the number of isolates decreased at that length of storage. A total of 146 strains from 10 genera and *Mycelia sterilia* were identified in barley stored for 3 months. There were negligible differences in the genera representation of filamentous fungi, but the highest number of isolates was found in the Laudis variety. *Alternaria* was the most frequently observed/isolated genus with 45 isolates and RD of 31% of all the isolates found. *Cladosporium* and *Rhizopus* were the second predominant genera with RD of 19%, each.

After 6 months of barley grain storage, the number of microfungi increased slightly to a total of 149 isolates with a comparable representation of 10 genera and *Mycelia sterilia* to those detected after 3 months of storage. In all samples, the genera *Alternaria, Arthrinium, Epicoccum, Fusarium, Penicillium,* and *Rhizopus* were detected. The RD of genus *Alternaria* was the highest (44%). From five distinct *Penicillium* species detected, the most frequent and the most abundant was *P. griseofulvum*. The numbers of genera of filamentous microscopic fungi detected in each barley variety reached their maximum at this storage period: 9 genera and *Mycelia sterilia* for the Wintmalt variety and 8 genera for the Laudis and Kangoo varieties, respectively.

The barley grain samples were contaminated comparably less with the filamentous microfungi at 9 months of storage (133 isolates). On the other hand, the most fungal genera (11) were detected in these samples. The highest RD was observed for the genus *Alternaria* (40%), followed by *Cladosporium* (19%), and *Penicillium* (10%). The highest amount of *Alternaria* isolates (24) was again isolated from the Laudis variety. Genera *Alternaria*, *Arthrinium*, *Cladosporium*, *Epicoccum,* and *Penicillium* were detected in all samples at 9 months of storage.

The data in Table 2 also show that, from the barley grains stored in silo for a period of 3 to 9 months, 13 fungal genera and *Mycelia sterilia* were identified (428 isolates). *Alternaria*, *Arthrinium,* and *Penicillium* were the main components of the mycobiota from 3 samples (100% IF), followed by *Epicoccum* (89%), *Cladosporium*, *Fusarium*, and *Rhizopus* (78%, each). *Alternaria* was the most abundantly detected genus (163 isolates) with RD of 38%. The isolated *Penicillium* consisted of eight species. *Penicillium brevicompactum* and *P. griseofulvum* showed the highest IF (33%) and RD (13%) of all the isolates from this genus.

Analysis of the detected filamentous fungi contaminants of barley showed that the Laudis barley variety had slightly higher number of isolates (47) and of genera (9) in comparison with Kangoo or Wintmalt varieties (Table 3). *Alternaria*, *Arthrinium*, *Cladosporium*, *Fusarium,* and *Penicillium* were detected in all samples. The most abundant genus was *Alternaria* (39%), followed by *Fusarium* (15%), and *Arthrinium* (12%). During the survey, 7 isolates of 4 *Penicillium* species (*P. crustosum*, *P. glabrum*, *P. griseofulvum*, and *P. chrysogenum)* were isolated and identified.

In the samples stored in floor warehouse, comparatively fewer filamentous fungi contaminants were detected than in the samples stored in grain storage silo. A total of 134 strains from 10 genera and *Mycelia sterilia* were identified at 3 months of storage (Table 4). 

A total of 89 isolates from 8 genera and *Mycelia sterilia* were isolated from samples stored for 6 months. *Alternaria* and *Penicillium* were detected in all samples. Alternaria was the most abundant genus (55%), especially in the Laudis variety. Compared with samples stored 6 months, a decrease of filamentous fungi detected was recorded at 9 months of storage in both types of storage. A total of 84 strains from 7 genera were identified from barley stored in floor warehouse for 9 months. *Alternaria* and *Arthrinium* were detected in all the samples examined. The occurrence of *Penicillium* spp. was generally low, but rich on species: *P. griseofulvum*, *P. hordei*, *P. chrysogenum*, and *P. raistrickii.* The lowest qualitative (4 genera) and quantitative (19 isolates) representation of microfungi was isolated from the Kangoo variety (Table 3). The most abundant genera were *Alternaria* and *Arthrinium* (42%, each).

A total of 307 strains from 10 genera and *Mycelia sterilia* were identified from the barley grain samples stored in floor warehouse over a period of 3 to 9 months. The most frequent were *Alternaria* (100% IF), *Penicillium* (89%), *Arthrinium* (78%), and *Epicoccum* (67%). The most abundant genera found by descending order were *Alternaria* (44% RD), *Arthrinium* (17%), and *Fusarium* (7%). The isolated *Penicillium* strains included/encompassed 7 species, of which *Penicillium griseofulvum* achieved the highest IF (33%) and RD (23%).

### 2.2. Endogenous Mycobiota of Malt

Samples of barley grains fresh from the harvest and before storage, and of barley grains stored in silo for 3, 6, and 9 months were malted and these samples were examined for endogenous mycobiota by plating method with surface disinfection. The filamentous fungi identified in malt are indicated in Table 5, and their IF and RD are presented in Table 6. The total count of microscopic fungi isolated from grains malted directly after harvest was the highest (112) with 7 genera and *Mycelia sterilia*, compared to malts prepared from barley stored in silo. *Alternaria, Epicoccum,* and *Fusarium* were the dominant fungal genera detected in the control malt. *Alternaria* was the most abundant occurring genusthere, with RD of 45%, but their presence in the Laudis and Kangoo malt were lower compared to the non-malted barley grain samples. The second abundant genus was *Epicoccum* (17%), followed by *Fusarium* (16%). Interestingly, the Wintmalt variety had the highest number of isolates. We isolated toxicogenic *Penicillium griseofulvum* and *P. chrysogenum* from the Wintmalt variety only.

The endogenous mycobiome in malts prepared from barley stored for 3 months included 63 isolates from 8 genera and *Mycelia sterilia*. *Alternaria* was the most abundant occurring genus with 22 isolates and RD 35% of all the isolates found, followed by *Mucor* (32% RD). Their IF was the highest in the Wintmalt variety.

Isolation of fungi from malts from surface-sterilized barley grains stored for 6 months resulted in collecting of 94 fungal isolates from 8 genera and *Mycelia sterilia*. *Alternaria, Cladosporium*, *Epicoccum*, and *Mucor* were the predominant genera in all 3 samples of malt. *Alternaria* scored the highest RD (65%) and was the main component of Wintmalt and Laudis malt mycobiota.

The overall mycological colonization of 3 tested malts from barley stored for 9 months was the lowest. Within 54 isolates of filamentous fungi, the most abundant were *Alternaria* (35% RD) and *Rhizopus* (28% RD). *Alternaria*, *Cladosporium*, *Epicoccum*, *Mucor*, and *Rhizopus* were more frequently isolated from malts (67% IF), and the remaining 2 genera and *Mycelia sterilia* were found only in one sample of malt.

A total of 211 isolates from 10 genera and *Mycelia sterilia* were identified from malts prepared from barley stored in grain storage silo for 3 to 9 months. The most frequent were *Alternaria* (89% IF), *Mucor* (78% IF), *Cladosporium,* and *Epicoccum* (67% IF, each). The most abundant genera found were *Alternaria* (48% RD), *Mucor* (14%), and *Rhizopus* (8%).

All the previous fungal genera except *Absidia* were also isolated from malted barley grain samples stored in floor warehouse (Table 7). Nevertheless, their relative abundances varied (Table 8). Isolates from malted barley stored in floor warehouse for 3 months included 93 strains from 7 genera of filamentous fungi and *Mycelia sterilia* with 100% isolation frequency for *Alternaria* and *Mucor*. *Alternaria* isolates predominated (55% RD), followed by *Cladosporium* (12% RD), *Mucor*, and *Penicillium* (10% RD, each). The incidence of *Penicillium* isolates revealed the occurrence of 4 different *Penicillium* species with maximum relative density for *P. corylophilum* (44% RD) of all the *Penicillium* isolates. Samples of malt prepared from barley grains stored for 6 months in floor warehouse had the lowest number (26) and genera (3) of filamentous fungi. The highest RD was again reached by the genus *Alternaria* (81%) which was present in all 3 samples. Microfungi in malt samples from barley grains stored in floor warehouse for 9 months included 73 isolates from 8 genera. The most common fungal genus was *Epicoccum* (100% IF) and the highest RD was reached by the genera *Rhizopus* (29%), followed by *Alternaria* and *Penicillium* (19% RD, each). Overall, 192 isolates from 10 genera and *Mycelia sterillia* were identified in malts prepared from barley stored in floor warehouse over a period of 3 to 9 months, very similar to the malt samples from grain stored in silo. The most frequent were *Alternaria* (78% IF), *Epicoccum, Mucor* (56% IF, each), *Cladosporium,* and *Penicillium* (44% IF, each). The most abundant genera found were *Alternaria* (45% RD), *Penicillium,* and *Rhizopus* (12% RD, each). From the 23 *Penicillium* isolates identified, the most frequent was *P. corylophilum* (22% IF) with the highest RD being 17% of all the *Penicillium* isolates.

Thirteen different genera and *Mycelia sterilia* were isolated from 3 varieties of freshly harvested barley grains before storage (Table 9). The total filamentous fungal count on control grains before storage ranged from 3.6 × 10^2^ to 3.6 × 10^3^ CFU/g and the total yeast count from 1.3 × 10^3^ to 9.1 × 10^4^ CFU/g.

Gradually, at the 6th month of storage, the grains mycobiota increased in incidence of the *Penicillium* species, due to storage conditions such as high atmospheric humidity. The barley grains associated mycobiota consisted of various species of fungi such as *Alternaria*, *Arthrinium*, *Aspergillus*, *Cladosporium*, *Fusarium*, *Penicillium*, *Ulocladium*, *Mycelia sterilia*, *Candida*, *Issatchenkia*, *Rhodotorula*, *Sporobolomyces*, and *Wickerhamomyces* belonging with predominance to the *Penicillium, Aspergillus*, and *Rhodotorula* genera. Yeasts from the analyzed samples were identified using MALDI-TOF MS Biotyper. *Penicillium* strains were isolated from all samples. The genus *Penicillium* was represented by eight species: *P. aurantiogriseum, P. canescens, P. citrinum, P. glabrum, P. griseofulvum, P. polonicum,* and *P. raistrickii*. At the 6th month of storage, the yeast abundance reached its maximum in all monitored varieties. The highest increase was observed in the Laudis variety with the yeast population ranging from 1.3 × 10^3^ CFU/g from the freshly harvested barley grain samples to 2.4 × 10^7^ CFU/g. A slight decrease compared to this maximum count in yeasts and filamentous fungi was observed at the 9th month of storage. *Cladosporium* was the predominant genus, followed by *Alternaria, Arthrinium, Aspergillus, Rhizopus,* and *Rhodotorula*. *Penicillium* was comparably less frequently isolated. *Rhodotorula* isolates occurred in the barley varieties Laudis and Kangoo and *Candida* and *Sporobolomyces* in the variety Wintmalt throughout the storage period in both types of storage.

The studies of barley grains stored in floor warehouse revealed that the initial yeasts count increased at 3rd and 6th months of storage and slightly decreased at 9th months of storage. At the 6th month of storage, the yeast abundance reached maximum in all monitored varieties, and in the 9th month decreased. The yeast population isolated from barley grains ranged from 1.3 × 10^3^ CFU/g from freshly harvested samples to 1.1 × 10^6^ CFU/g in the Wintmalt variety stored for 6 months. Samples were less contaminated with fungi. At the 3rd month of storage, the microfungi abundance reached maximum and included *Alternaria, Arthrinium, Aspergillus, Cladosporium, Epicoccum, Fusarium, Penicillium,* and *Mycelia sterilia*. In the Kangoo variety stored for 3 months, fewer filamentous fungi were detected than from the freshly harvested samples. Saprophytic fungi such as *Arthrinium* and *Aspergillus*, isolated from samples at 3 months of storage, were not detected at all in the samples at 6th and 9th months of storage, and in these samples, only yeasts were observed. We did not isolate any microfungi from malting barley of the Wintmalt variety after 9 months of storage. Strains of *Penicillium* forming a dominant part of fungal isolates from barley stored in silo were less often isolated from samples stored in floor warehouse, mainly in the Wintmalt variety.

Analysis of the samples of malted barley grain revealed that malting conditions were favorable for fungal growth (Table 10). Changes in the abundance of fungi in malted barley grain were evident. In the malt from freshly harvested grains, the total yeast count was from 2.2 × 10^3^ to 4.5 × 10^3^ CFU/g and filamentous fungi from 1.1 × 10^3^ to 2.2 × 10^3^ CFU/g. We detected 6 genera of filamentous fungi and 3 genera of ascomycetous yeasts and 2 genera of basidiomycetous yeasts from the malting trials. *Aspergillus* and *Cladosporium* were the dominant genera in the samples of barley after malting.

In this work, the initial fungal counts during storage gradually increased in malt prepared from barley at 3rd, 6th, and 9th month under both types of storage. *Alternaria* formed a dominant part of filamentous fungal isolates from malted barley grains stored for 9 months in silo, followed by *Penicillium*. At the end of the 9th month, *Mucor* dominated in malted barley grains stored in floor warehouse, where *Alternaria* was the dominant genus at 3 months of storage; at 6th month of storage, there were *Alternaria, Penicillium,* and *Mucor* and at 9th month of storage, only *Penicillium* dominated. *Rhodotorula* spp. formed a dominant part of yeast isolates in malt prepared from the Laudis and Kangoo varieties and *Candida saitoana* and *Sporobolomyces roseus* dominated in malt prepared from the Wintmalt variety throughout the whole storage period in both types of storage.

### 2.3. Toxigenicity of Penicillium Species

The toxigenic profile of the *Penicillium* isolates representing *P. crustosum, P. expansum, P. griseofulvum, P. hordei, P. chrysogenum,* and *P. raistrickii* from barley grains and malt is shown in Table 11. In total, 14 isolates representing 3 potentially toxigenic species were isolated from barley and tested for their toxigenic ability. Positive toxigenicity was detected in *P. chrysogenum* and *P. raistrickii*. *Penicillium griseofulvum* produced griseofulvin (G), cyclopiazonic acid (CPA), and patulin (P, 5 out of 6 isolates screened) but did not produce roquefortin C (RC). In total, 25 endogenous isolates representing 6 potentially toxigenic species from barley were tested for their toxigenic ability. Positive toxigenicity was detected in *P. hordei* and *P. raistrickii. Penicillium crustosum* produced penitrem A, but did not produce RC. *Penicillium expansum* produced P, G, but not RC. RC production was also tested in 8 isolates of *P. griseofulvum*, but it was not detected. On the other hand, *Penicillium griseofulvum* produced P (6 out of 8 isolates tested), G (7 out of 8 isolates), and CPA (7 out of 8 isolates). No toxigenicity was detected in *P. chrysogenum* isolated from malt or the endogenous mycobiota of malt. *Penicillium griseofulvum* from the endogenous mycobiota of malt produced CPA, G, and P (1 out of 2) and did not produce RC. Out of 47 isolates tested, 79% produced at least one mycotoxin as revealed by the method used here.

## 3. Discussion

Papadopoulou et al. [22] reported that the fungi found most frequently on barley include *Fusarium, Aspergillus, Penicillium, Alternaria,* and *Cladosporium*. All but *Penicillium* were isolated from our samples, with *Aspergillus* and *Cladosporium* with low incidence. The most frequently isolated fungal species on 43 malting barley samples from Italy belonged to the genera *Alternaria* and *Fusarium* [23]. The *Fusarium* species belongs to the most toxigenic fungi in the northern temperate regions [24]. Barley contaminated with these pathogenic fungi is negatively influenced during vegetation, can show reduced germination capacity and quality of malting grain, can show losses in yield, decrease in the quality of malt and beer, and result in non-safe products [20,25]. One known side effect of *Fusarium* fungi presence is gushing, with occasionally great economic losses for breweries from the over-foaming of beer. As such, one of the consequences of barley grain contamination can be the loss of customers [26]. Biliková and Hudec [27] found out that the use of corn as a pre-crop could increase the existence of *Fusarium* head blight of barley as well as in the manufactured beer. The production of harmful metabolites—mycotoxins that have great impact on human and animal health—poses an even greater risk [20]. Krasauskas [24] reported that some species of *Alternaria* spp. on barley grain samples were dominant before storage in Lithuania, which is in line with our results. *Alternaria* is one of the major mycotoxigenic fungal genera with more than 70 reported toxic metabolites. *Alternaria* is a fungal genus encompassing both saprophytic and pathogenic species, widespread in nature. The highest levels of *Alternaria* mycotoxin tenuazonic acid were detected in cereals, followed by beer; the highest levels of alternariol and alternariol methyl ether were found in lentils, oilseeds, tomatoes, carrots, juices, wines, and cereals [28]. Several other fungi were isolated from the Lithuanian samples of barley grain: *Aspergillus niger, A. flavus, Cladosporium herbarum, Fusarium culmorum, F. poae*, *F. equiseti*, and *Rhizopus nigricans.* These fungi were also isolated from our samples. Krasauskas [24] mentioned other microfungi not isolated in our experiment: *Acremonium strictum, Botrytis cinerea, Drechslera sorokiniana, Mucor* spp., *Nigrospora sphaerica, Penicillium* spp., *Rhizoctonia* spp., *Stemphylium* spp., *Trichoderma* spp., and *Verticillium alboatrum*.

Birck et al. [29] analyzed fungal contamination in wheat grain during storage for 180 days. *Fusarium* spp. was the most prevalent fungus after harvest as well as after 30 days of storage, and the counts decreased gradually until the end of the storage period. *Aspergillus, Fusarium,* and *Penicillium* were found in 96.7%, 46.7%, and 80.0% of wheat samples, respectively, after 180 days of storage. These results are similar to those that we accrued in our study. *Fusarium* genera were one of the most abundant genera after harvest, but their count gradually decreased. The genera *Fusarium, Alternaria, Cladosporium,* and *Epicoccum* belong to common fungi on cereal grains in the field [30]. The micromycetes of *Fusarium* spp. are described as ‘‘field fungi”, but in some cases, they can grow during storage as well [20,31]. *Alternaria* was the most abundant occurring genus (38% RD) in our samples. Krnjaja et al. [32] focused on mycobiota of maize from Serbia. Mycological analyses found the presence of *Aspergillus, Fusarium,* and *Penicillium* on both freshly harvested and stored grains. The predominant species were different in each stage of processing. A similar observation was made for maize from various locations in Romania. The samples analyzed immediately after the harvest showed predominance species of *Alternaria*, followed by species of *Cladosporium* and *Penicillium.* The maize grains after 120 days of storage contained mycobiota composed of various fungi with predominance of *Fusarium, Aspegillus,* and *Penicillum* [33]. *Fusarium* does not survive on dry stored barley as long as *Alternaria* does; the spores of *Alternaria* may remain viable for a number of years [6]. This explains the fact that if barley is properly stored, mainly *Alternaria* species are detected.

*Penicillium* is characterized as xerophilic with a short time span of the asexual reproduction. Xerophilic species *Penicillium corylophilum* takes advantage of the possibility to disperse to open and available habitats where rapid asexual life cycle takes place. The competition advantage for *P. corylophilum* probably does not persist if the relative humidity increases. On the contrary, under a relatively higher humidity, probably more species combating for the same open niche could be found. The presence of these species poses did not confirm inhalation hazards and dermal inoculation hazard as well [34]. *Penicillium corylophilum* was the most frequent species (67% IF) isolated with high relative density (44%) of all the *Penicillium* isolates of endogenous colonization from malted barley grains stored for 3 months.

The total filamentous fungal count on control grains (freshly harvested, before storage) ranged from 3.6 × 10^2^ to 3.6 × 10^3^ CFU/g and the total yeast count from 1.3 × 10^3^ to 9.1 × 10^4^ CFU/g. The number of fungi propagules was comparatively lower, from 6.2 up to 9.0 CFU/g in barley grain before storage from Lithuania [24]. The malting barley grains collected from Southern Brazil also presented a low count of fungal colonies, with values ranging from 10.5 to 50 CFU/g (mean: 28 ± 25.5 CFU/g) [35]. In total, 9 fungal isolates were identified from 4 distinct genera: *Fusarium* (6), *Penicillium* (1), *Alternaria* (1), and *Rhizopus* (1). Climatic conditions (temperature and high humidity) in Southern Brazil may influence the contamination by *F. graminearum* and, consequently, maximize the production of deoxynivalenol. Samples colonized by *Fusarium*, especially *F. graminearum* and *F. verticillioides*, were predominant in the 19 isolates, with 13 belonging to *F. graminearum* and 6 belonging to *F. verticillioides*. The incidence of both species was 26% and 12%, respectively. There was high contamination of barley with this fungi found in other experiments [31,35], especially for the species *F. graminearum*. Their results are in line with our study and confirmed that *Fusarium* was detected from all 3 barley varieties analyzed. The majority (61%) of brewer’s grain samples collected from a major Argentinean brewery and 83% of the malted barley (malt) had a total fungal count >1 × 10^4^ CFU/g [36]. In our study, it was comparatively lower, with 48% of brewer’s grain samples and 79% of the malt samples that had this count. Yeasts were isolated from all brewer’s grain and malt samples [36], as in our study. Genera containing some of the most important toxicogenic species—*Fusarium* spp., *Aspergillus* spp., *Penicillium* spp., and *Alternaria* spp.—were isolated from the analyzed samples, along with other environmental saprophytic fungi such as *Geotrichum* spp., *Mucorales,* and *Cladosporium* spp. All mentioned fungal genera were isolated from our samples as well.

The main factor responsible for limiting the development of microscopic fungi is low humidity in grain. In the temperate climate zone, the majority of micromycetes recorded on grain are mesophiles. The most suitable temperature for their development is between 15 to 30 °C [12], although some micromycetes species are able to develop at temperatures below 0 °C (*Cladosporium herbarum, Fusarium nivale, Fusarium avenaceum*) [37]. Humidity of our grain was below 15% and the air temperature during storage fluctuated below 25 °C. The number of fungi propagules increased under the storage conditions and fluctuated from 8.3 up to 15.2 CFU/g [24]. The number of fungi propagules was comparatively higher than in barley ears before the harvest (the dust rising from the soil was carried into the tank of the combine harvester together with grain during the harvest). Fungi isolated from dried and stored barley grain in storage silo included *Arthrobotrys oligospora, Aspergillus niger, A flavus, Aspergillus oryzae, Fusarium sporotrichioides, F. poae, Penicillium expansum, P. verrucosum, P. viridicatum,* and *Scopulariopsis brevicaulis*. The dominant species on samples of barley grain from a storage silo were *Aspergillus* spp. and *Scopulariopsis* spp. [24]. Przybylska-Balcerek et al. [38] reported that the content of microscopic fungi in all 44 barley grain samples from cereal silo was very low from October to November and it ranged from 1.81 to 2.19 log CFU/g. Fungi from the genus *Fusarium* were identified among three most frequently found genera of microscopic fungi, which is in contrast to our results.

The initial yeasts count increased at the 3rd and 6th month of both types of storage and slightly decreased at the 9th month of storage. The quantitative representation of filamentous microscopic fungi was lower compared to yeasts. Some encapsulated yeasts were able to survive during the long-term storage of barley. On the other hand, the number of bacteria and filamentous fungi associated with kernels decreased [21,39].

The most important group on samples of barley after malting was genus *Fusarium,* which was quite frequent. Krasauskas [24] reported the intensive growth of *Fusarium* during the steeping, even when the barley from the storage silo initially had only a low level of *Fusarium* contamination. *Geotrichum* spp. and other yeasts, *Mucor* spp. and *Fusarium* spp., were dominant species on samples of barley after malting.

The most significant mycotoxins found in cereals are produced by *Fusarium* (trichothecenes, zearalenone, fumonisins), *Aspergillus* (aflatoxins, ochratoxin A), and *Penicillium* (ochratoxin A). The range of the mycotoxins present depends on the locality, weather, crop variety, as well as the growing year [40]. A recent multi-annual global survey [41] concluded that deoxynivalenol was the abundant mycotoxin in the barley samples collected from different countries, followed by zearalenone and T-2/HT-2 toxins. The presence of the potentially toxigenic fungus *Aspergillus* was very low (<1%). The genus *Fusarium* occurred mainly in the field, and we recorded its decreased frequency during storage. The occurrence of the genus *Penicillium* was the highest in warehouses, but its RD did not exceed 7%. The mycotoxin levels declined during malting; however, their production can increase during germination as the warm, humid environment of a malthouse is a suitable environment for mold growth. The further mycotoxin production is possible during kilning. High temperature can cause the elimination of fungal growth, but an increase in temperature during initial stages of kilning can stimulate an increased mycotoxin production in some fungal species [19]. We observed an increase only in the genus *Penicillium* in malt during the storage of barley. Ochratoxin A (OTA) is one of the most important mycotoxin in cereals. There was no isolated *Penicillium verrucosum*, as a potential producer of OTA. Our results showed that the *Penicillium* species commonly isolated from barley and malt could be a potential source of the mycotoxins patulin, griseofulvin, and cyclopiazonic acid under in vitro conditions.

## 4. Materials and Methods

### 4.1. Samples

Contamination of barley grains with micromycetes during different periods of storage was investigated from 2018 to 2019. Grain samples of barley were taken for mycological analysis both directly from the fields of the farmers who grew it for malt producers and from the grains stored in floor warehouse and grain silo in Maltery Heineken Slovensko Sladovne in Hurbanovo, Slovak Republic.

Silos—Each silo consists of several cells; in our case, it was 21 hexagonal square chambers with a capacity of 1000 tons and with a conical bottom (45°) to facilitate emptying. Each cell in the silo was equipped with 10 thermometers placed along its entire height. The dedicated program then calculated an average value from 10 recorded temperatures. The program works on the principle of a traffic light controller and according to the value of the recorded average temperature, it is marked either in green (suitable temperature up to 20 °C), yellow (20 to 25 °C), or red (above 25 °C), in which case it is necessary to intervene. In the event of deteriorating conditions or as part of regular relocations, every 2 to 3 months, transfer of the stored grain to another free cell is performed. The emptied cell is then cleaned and gassed before refilling to limit the multiplication of microbial contaminants and pests.

Floor warehouse—The grain was stored on the floor in layers of 4 to 5 m, with temperatures 15 to 22 °C. The storage facility was built of concrete halls, usually with recessed grate ducts enabling air distribution during active ventilation. This type of storage has a system of perforated pipes placed on the floor, which use fans to regulate the aeration in order to remove heat, carbon dioxide, and thus reduce humidity. Parts with different humidity should not be mixed during storage, because in places with higher humidity, a quicker heat formation might occur accompanied with material losses and also with possible microbial contamination.

Barley samples (15 kg each) were placed in perforated bags that were stored in grain silo cells, along with other stored barley. With this procedure, we were able to imitate the process of mass grain storage. Once every 3 months (until the 9th month), we removed these bags and took out the required amount of barley for analysis (3 kg). Then, we returned the bags back into the cells. Temperatures in floor warehouse during the storage were in the range 15 to 22 °C and in the silos from 15 to 25 °C. Humidity of grain was below 15%. The study was conducted using 3 types of malting barley Laudis and Kangoo (two-row spring malting barley variety), grown in the Levice region, and Wintmalt (two-row winter malting barley variety) grown in the Zlaté Moravce region in Slovak Republic. Samples were sent to the laboratory and 1 kg from each sample was malted.

### 4.2. Micro-Malting Procedure

Malt processing was carried out in the center of AgroBioTech in Slovak University of Agriculture in Nitra, Slovakia. Samples (1 kg each) of barley grain were malted in laboratory micromalting plant 4 times, from freshly harvested barley (0 weeks of storage), 3 months, 6 months, and 9 months after start of the storage trial.

Steeping—1st day water for 5 h, 2nd day water for 4 h, full steeped on water content 45.5%. Between the two water phases, air rests were performed, where barley samples were aerated with fresh air in the steeping box.

Germination was performed at a malt temperature of 15 to 16 °C and proceeded at continuous aerating with fresh conditioned air. Total time of steeping and germination was six days.

The kilning process was performed on an electrically heated one-floor kiln, with a gentle and gradual increase in temperature up to 80 °C for 4 h.

### 4.3. Mycological Analysis of Barley and Malt

A total of 50 grains from each sample were surface disinfected in 1% NaClO for 1 min according methods of Magnoli et al. [42] and rinsed 3 times by submersion in sterile distilled water (total amount 1 L), dried, plated in DRBC (Dichloran Rose Bengal Chloramphenicol agar medium; MERCK, Germany), and incubated at 25 ± 1 °C in the dark for 7 days. Endogenous mycobiota was determined in this way.

Dilution plating method (surface-spread method) was used for colony counting. Ten grams of each grain sample were individually homogenized in 90 mL of physiological saline solution (0.89%, MERCK, Germany) for 30 min. Serial decimal dilutions up to 10^−5^ were made and 0.1 mL were inoculated in triplicates onto Petri dishes with Dichloran Rose-Bengal Chloramphenicol (DRBC) medium. The dishes were incubated in the dark in thermostat (BT 120, Praha, Czech Republic) for 7 days, at the temperature of 25 ± 1 °C.

Fungi were identified according to morphological and microscopic characteristics [10,43,44].

Colonies representative of *Penicillium* were transferred for subculturing to plates containing CYA (Czapek yeast agar) [44], MEA (Malt extract agar) [44], YES (Yeast Extract agar) [44], and CREA (Creatine-Sucrose agar) [44] media. Species identification was carried out according to available taxonomic keys and guides [10,43,44,45].

The results were expressed as isolation frequency of the fungal genera (IF; defined as the percentage of samples in which each genus or species was present in relation to the total number of samples) and relative density (RD; the relative percentage of isolates of each genus or species, occurring in the analyzed sample) [46]. These values were calculated according to González et al. [47] as follows:

IF (%) = (ns/N) × 100

RD (%) = (ni/Ni) × 100

ns—number of samples with a given species or genus; N—total number of samples; ni—number of isolates of a given species or genus; Ni—total number of isolated fungi.

### 4.4. Mycotoxins Detection

Toxigenicity assays of selected isolates were performed under in vitro conditions using thin layer chromatography (TLC) [48], modified by Labuda and Tančinová [49]—Griseofulvin and patulin assays were carried out on YES agar as extracellular metabolites and roquefortin C, cyclopiazonic acid, and penitrem A on CYA agar as intracellular metabolites. A few pieces of mycelium with approximate size 5 × 5 mm were cut from colonies and placed in an Eppendorf tube with 500 μL of chloroform:methanol (2:1; *v/v*; Reachem, Bratislava-Petržalka, Slovak Republic). The content of the tubes was stirred for 5 min by Vortex G-560E (Scientific Industries, Bohemia, NY, USA). The extracts were screened for mycotoxins contamination by applying small spots of 30 μL of each extract and 10 μL standard solutions (Sigma, Taufkirchen, Germany) on a silica gel 60 TLC aluminum sheet (20 × 20 cm; Alugram^®^ SIL G, Macherey—Nagel, Düren, Germany) and developed with TEF solvent mobile phase (toluene:ethyl acetate:formic acid, 5:4:1 *v/v/v*; toluene—Mikrochem, Pezinok, Slovak Republic; ethyl acetate and formic acid—Slavus, Bratislava, Slovak Republic). Thereafter, the plate was air-dried. Roquefortin C was visible after spraying with Ce(SO_4_)_2_ × 4 H_2_O as an orange spot, patulin by spraying with 0.5% methylbenzothiazolone hydrochloride (MBTH), (Merck, Darmstadt, Germany) in methanol and heating at 130 °C for 8 min as a yellow–orange spot. Cyclopiazonic acid was visible directly in daylight after spraying with the Ehrlich reagent as a violet-tailed spot. Penitrem A after spraying with 20% AlCl_3_ in 60% ethanol and heating at 130 °C for 8 min as a dark blue spot. Griseofulvin was visualized directly under UV light (Krüss Optronic, Hamburg, Germany) with a wavelength of 365 nm as a blue spot.

### 4.5. Mass Spectrometry Identification of Yeast Isolates

Qualitative determination of yeasts was carried out using the MALDI-TOF Mass Spectrometry device (Bruker Daltonics, Bremen, Germany). The isolates of yeasts were sub-cultured on Tryptone soya agar (TSA, Oxoid, Basingstoke, UK) at 30 °C for 24 h. The criteria for reliable identification were a score of ≥2.0 at the species level [50]. Isolates were put in 300 μL of distilled water and 900 μL of ethanol, and the suspension centrifuged for 2 min at 14,000 rpm. The pellet was centrifuged repeatedly and allowed to dry. Then, 30 μL of 70% formic acid and 30 μL of acetonitrile were added to the pellet. Tubes were centrifuged for 2 min at 14,000 rpm and 1 μL of the supernatant was used for MALDI identification. Once dry, every spot was overlaid with 1 μL of an HCCA (α-Cyano-4-hydroxycinnamic acid) matrix and left to dry at room temperature before analysis. Generated spectra were analyzed on a MALDI-TOF Microflex LT (Bruker Daltonics, Bremen, Germany) instrument using Flex Control 3.4 software and Biotyper Realtime Classification 3.1 with BC-specific software.

### 4.6. Statistical Analysis

For every test, three independent repeated measurements were done, unless stated otherwise. All measurements were compared using one-way analysis of variance independently for each dependent variable. Post-hoc Tukey HSD (honest significant difference) multiple comparison tests were used to identify statistically homogeneous subsets at α = 0.05. Statistical analysis of the data was performed with the Statistica 13 (Dell Software Inc., Round Rock, TX, USA) software.

## 5. Conclusions

The present study contributes to the knowledge of mycobiota in Slovakian malting barley and malt, and to the presence of toxigenic fungi during the storage period. Based on the obtained results, from the potentially toxigenic fungi *Alternaria, Aspergillus, Fusarium,* and *Penicillium,* only the field fungi *Alternaria* and *Fusarium* were present in the harvested barley grain samples and all of them were present in stored grain, with the highest incident for *Alternaria*, followed by *Penicillium, Fusarium,* and *Aspergillus*. This study also provided a clear indication of the yeast diversity in the malt ecosystem. In this work, *Rhodotorula* spp. was the most prevalent genus isolated from pre- and post-stored malt samples. The quantity of fungi in grain conversion technology from the storage to the malting process was increasing. Species of the genus *Penicillium* had higher incidence in stored samples compared to malt. This study also provides new insights into the diversity of *Penicillium* species isolated from Slovakian barley: *P. crustosum, P. expansum, P. griseofulvum, P. hordei, P. chrysogenum,* and *P. raistrickii*, with the predominant species *Penicillium griseofulvum,* were all detected. Fungi were isolated from healthy grain up to 9 months of storage. However, the number of samples from which the fungi were isolated generally decreased with increasing storage time. For this reason, constant supervision and monitoring of potential toxigenic fungi or mycotoxins occurrence in barley and malt pre- and post-harvest, as well as application of preventive measures are very important to reduce the related risks to consumer health.

## Figures and Tables

**Table 1:** Number of microfungi from endogenous mycobiota of barley stored in silo for various periods.

Fungal Taxa/ Varieties	after Harvest			after 3 Months			after 6 Months			after 9 Months		
	L	K	W	L	K	W	L	K	W	L	K	W
*Alternaria*	44	19	19	11	11	23	26	16	23	24	10	19
*Arthrinium*	4	9	4	11	3	2	7	1	3	4	4	1
*Aspergillus*		1			2		2	2			2	1
*Bipolaris*										3		
*Cladosporium*		1		27		1	2		2	8	4	13
*Epicoccum*	6	3	2	5		4	4	4	4	7	2	1
*Fusarium*	15	4		7	1		10	4	2	1		1
*Geotrichum*						2			1			
*Mucor*					1						1	
*Penicillium*				2	1	2	5	2	5	4	5	5
*P. aurantiogriseum*							1		1			
*P. brevicompactum*							1			1		2
*P. crustosum*				1								
*P. expansum*												3
*P. glabrum*									1			
*P. griseofulvum*				1				1	2			
*P. chrysogenum*											2	
*P. raistrickii*								1	1	1		
*Penicillium* spp.					1	2	3			2	3	
*Rhizopus*		1	5	2	19	7	2	14	1		11	
*Sordaria*	1									1		
*Stemphylium*								1	3			
*Mycelia sterilia*	2		2		2				3			
**Total**	72	38	32	65	40	41	58	44	47	52	39	41

L—Laudis, K—Kangoo, W—Wintmalt.

**Table 2:** Number of isolates, isolation frequency (%), and relative density (%) from endogenous mycobiota of barley depending on the storage time from silos.

Fungal Taxa/Varieties	after Harvest			after 3 Months			after 6 Months			after 9 Months			from 3–9 Months		
	n	IF	RD	n	IF	RD	n	IF	RD	n	IF	RD	n	IF	RD
*Alternaria*	82	100	58	45	100	31	65	100	44	53	100	40	163	100	38
*Arthrinium*	17	100	12	16	100	11	11	100	7	9	100	7	36	100	8
*Aspergillus*	1	33	<1	2	33	2	4	67	3	4	67	3	10	55	2
*Bipolaris*										3	33	2	3	11	<1
*Cladosporium*	1	33	<1	28	67	19	4	67	3	25	100	19	57	78	13
*Epicoccum*	11	100	8	9	67	6	12	100	8	10	100	7	31	89	7
*Fusarium*	19	67	13	8	67	6	16	100	11	2	67	1.5	26	78	6
*Geotrichum*				2	33	1	1	33	<1				3	22	<1
*Mucor*				1	33	<1				1	33	<1	2	22	<1
*Penicillium*				5	100	3	12	100	8	14	100	10	31	100	7
*P. aurantiogriseum*							2	67	17				2	22	6
*P. brevicompactum*							1	33	8	3	67	21	4	33	13
*P. crustosum*				1	33	20							1	11	3
*P. expansum*										3	33	21	3	11	10
*P. glabrum*							1	33	8				1	11	3
*P. griseofulvum*				1	33	20	3	67	25				4	33	13
*P. chrysogenum*										2	33	14	2	11	6
*P. raistrickii*							2	67	17	1	33	7	3	33	10
*Penicillium* spp.				3	67	60	3	33	25	5	67	36	11	56	35
*Rhizopus*	6	67	4	28	100	19	17	100	11	11	33	8	56	78	13
*Sordaria*	1	33	<1							1	33	<1	1	11	<1
*Stemphylium*							4	67	3				4	22	<1
*Mycelia sterilia*	4	67	3	2	33	1	3	33	2				5	22	1
**Total**	142			146			149			133			428		

n—number of isolates; IF—isolation frequency, RD—relative density.

**Table 3:** Number of microfungi from endogenous mycobiota of barley depending on the storage time from floor warehouse.

Fungal Taxa/Varieties	after 3 Months			after 6 Months			after 9 Months		
	L	K	W	L	K	W	L	K	W
*Alternaria*	11	15	26	26	7	16	21	4	10
*Arthrinium*	11	4	1	1			10	13	12
*Aspergillus*			1		1				
*Cladosporium*	3	1	1		3		2		
*Epicoccum*	3	5		4		3	4		1
*Fusarium*	6	6	8			2			
*Mucor*	1								
*Penicillium*	4	2	1	3	1	2		1	3
*P. aurantiogriseum*				3					
*P. crustosum*	2								
*P. glabrum*		1							
*P. griseofulvum*	2					1			1
*P. hordei*									1
*P. chrysogenum*		1							1
*P. raistrickii*					1	1		1	
*Penicillium* spp.			1						
*Rhizopus*	2	6		1	3		1		
*Sordaria*	6						1	1	
*Mycelia sterilia*		4	6		9	7			
**Total**	47	43	44	35	24	30	39	19	26

L—Laudis, K—Kangoo, W—Wintmalt.

**Table 4:** Number of isolates, isolation frequency (%), and relative density (%) from endogenous mycobiota of barley grains stored in floor warehouse for 3, 6, or 9 months.

Fungal Taxa/Varieties	after 3 Months			after 6 Months			after 9 Months			from 3–9 Months		
	n	IF	RD	n	IF	RD	n	IF	RD	N	IF	RD
*Alternaria*	52	100	39	49	100	55	35	100	42	136	100	44
*Arthrinium*	16	100	12	1	33	1	35	100	42	52	78	17
*Aspergillus*	1	33	<1	1	33	1				2	22	<1
*Cladosporium*	5	100	4	3	33	3	2	33	2	10	56	3
*Epicoccum*	8	67	6	7	67	8	5	67	6	20	67	5
*Fusarium*	20	100	15	2	33	2				22	44	7
*Mucor*	1	33	<1							1	11	<1
*Penicillium*	7	100	5	6	100	7	4	67	5	17	89	5
*P. aurantiogriseum*				3	33	50				3	11	18
*P. crustosum*	2	33	29							2	11	12
*P. glabrum*	1	33	14							1	11	6
*P. griseofulvum*	2	33	29	1	33	17	1	33	25	4	33	23
*P. hordei*							1	33	25	1	11	6
*P. chrysogenum*	1	33	14				1	33	25	2	22	12
*P. raistrickii*				2	67	33	1	33	25	3	33	18
*Penicillium* spp.	1	33	14							1	11	6
*Rhizopus*	8	67	6	4	67	5	1	33	1	13	56	4
*Sordaria*	6	33	4.5				2	67	2	8	33	3
*Mycelia sterilia*	10	67	7.5	16	67	18				26	44	8
**Total**	134			89			84			307		

n—number of isolates; IF—isolation frequency; RD—relative density.

**Table 5:** Number of microfungi from endogenous mycobiota of malted barley grains directly after harvest or stored in silos.

Fungal Taxa/Varieties	after Harvest			after 3 Months			after 6 Months			after 9 Months		
	L	K	W	L	K	W	L	K	W	L	K	W
*Absidia*				2								
*Alternaria*	11	3	36	5	1	16	22	3	36	3		16
*Arthrinium*										4		
*Aspergillus*							1					
*Cladosporium*	3	9			4		2	3	2	2		1
*Epicoccum*	10	5	4			3	1	2	4	2		2
*Fusarium*	7	2	9	2	4				1			
*Geotrichum*	1											
*Mucor*	1	1		4		16	1	3	3	2	1	
*Penicillium*			2		1				2			4
*P. griseofulvum*			1						1			
*P. chrysogenum*			1									
*P. raistrickii*												3
*Penicillium* spp.					1				1			1
*Rhizopus*					1				1	7	8	
*Mycelia sterilia*	2	3	1	3		1	5	2				2
**Total**	35	23	54	16	11	36	32	13	49	20	9	25

L—Laudis, K—Kangoo, W—Wintmalt.

**Table 6:** Number of isolates, isolation frequency (%), and relative density (%) from endogenous mycobiota of malted barley grains directly after harvest or stored in silos.

Fungal Taxa	after Harvest			after 3 Months			after 6 Months			after 9 Months			from 3–9 Months		
	n	IF	RD	n	IF	RD	n	IF	RD	n	IF	RD	n	IF	RD
*Absidia*				2	33	3							2	11	<1
*Alternaria*	50	100	45	22	100	35	61	100	65	19	67	35	102	89	48
*Arthrinium*										4	33	7	4	11	2
*Aspergillus*							1	33	1				1	11	<1
*Cladosporium*	14	67	12	4	33	6	7	100	7	3	67	6	14	67	7
*Epicoccum*	19	100	17	3	33	5	7	100	7	4	67	7	14	67	7
*Fusarium*	18	100	16	6	67	9	1	33	1				7	33	3
*Geotrichum*	1	33	<1												
*Mucor*	2	67	2	20	67	32	7	100	7	3	67	6	30	78	14
*Penicillium*	2	33	2	1	33	2	2	33	2	4	33	7	7	33	3
*P. griseofulvum*	1	33					1	33					1	11	
*P. chrysogenum*	1	33													
*P. raistrickii*										3	33		3	11	
*Penicillium* spp.				1	33		1	33		1	33		3	33	
*Rhizopus*				1	33	2	1	33	1	15	67	28	17	44	8
*Mycelia sterilia*	6	100	5	4	67	6	7	67	7	2	33	4	13	56	6
**Total**	112			63			94			54			211		

n—number of isolates; IF—isolation frequency; RD—relative density.

**Table 7:** Number of microfungi from endogenous mycobiota of malted barley grains stored in floor warehouse.

Fungal Taxa/Varieties	after 3 Months			after 6 Months			after 9 Months		
	L	K	W	L	K	W	L	K	W
*Alternaria*	25	4	22	6	5	10			14
*Arthrinium*									4
*Aspergillus*									1
*Cladosporium*	10		1		1			2	
*Epicoccum*	4		2				2	2	3
*Fusarium*						1			
*Geotrichum*		3							
*Mucor*	1	2	6				2	8	
*Penicillium*	1	8					5	9	
*P. aurantiogriseum*		1							
*P. brevicompactum*								2	
*P. corylophilum*	1	3							
*P. hordei*		1							
*P. polonicum*		3							
*Penicillium* spp.							5	7	
*Rhizopus*		1					17	4	
*Mycelia sterilia*	2		1	2		1			
**Total**	43	18	32	8	6	12	26	25	22

L—Laudis, K—Kangoo, W—Wintmalt.

**Table 8:** Number of isolates, isolation frequency (%), and relative density (%) from endogenous mycobiota of malted barley grains directly stored in floor warehouse.

Fungal Taxa	after 3 Months			after 6 Months			after 9 Months			from 3–9 Months		
	n	IF	RD	n	IF	RD	n	IF	RD	n	IF	RD
*Alternaria*	51	100	55	21	100	81	14	33	19	86	78	45
*Arthrinium*							4	33	5	4	11	2
*Aspergillus*							1	33	1	1	11	<1
*Cladosporium*	11	67	12	1	33	4	2	33	3	14	44	7
*Epicoccum*	6	67	6				7	100	10	13	56	7
*Fusarium*				1	33	4				1	11	<1
*Geotrichum*	3	33	3							3	11	2
*Mucor*	9	100	10				10	67	14	19	56	10
*Penicillium*	9	67	10				14	67	19	23	44	12
*P. aurantiogriseum*	1	33	11							1	11	4
*P. brevicompactum*							2	33	14	2	11	9
*P. corylophilum*	4	67	44							4	22	17
*P. hordei*	1	33	11							1	11	4
*P. polonicum*	3	33	33							3	11	13
*Penicillium* spp.							12	67	86	12	22	52
*Rhizopus*	1	33	1				21	67	29	22	33	12
*Mycelia sterilia*	3	67	3	3	67	11				6	44	3
**Total**	93			26			73			192		

n—number of isolates; IF—isolation frequency; RD—relative density.

**Table 9:** Quantitative and qualitative composition of yeast and filamentous fungi from 3 varieties of barley depending on the time and type of storage.

Time of Storage	Varieties	Yeast log CFU/g	Fungi log CFU/g	Species
0	Laudis	1.3 × 10 ^B^	3.6 × 10 ^C^	*Cladosporium, Fusarium, Mucor, Mycelia sterilia, Ogataea polymorpha, Rhodotorula* spp., *Issatchenkia orientalis*
	Kangoo	3.2 × 10 ^B^	3.6 × 10 ^B^	*Aspergillus, Cladosporium, Fusarium, Candida vini, Filobasidium oeirense, Candida* spp., *Rhodotorula* spp., *Wickerhamomyces anomalus*
	Wintmalt	9.1 × 10 ^A^	1.3 × 10 ^B^	*Fusarium, Penicillium chrysogenum, Mucor, Mycelia sterilia, Diutina catenulata, Candida saitoana, Sporobolomyces roseus*
Storage silo				
3 months	Laudis	1.8 × 10 ^b,AB^	8.5 × 10 ^a,A^	*Aspergillus, Fusarium, Ulocladium, Rhodotorula* spp., *Issatchenkia orientalis*
	Kangoo	8.9 × 10 ^b,A^	9.0 × 10 ^a,A^	*Aspergillus, Eurotium* (now *Aspergillus*)*, Mucor, Penicillium aurantiogriseum, Rhodotorula* spp., *Wickerhamomyces anomalus*
	Wintmalt	1.4 × 10 ^a,B^	<1 × 10	*Cladosporium, P. citrinum, P. griseofulvum, Candida saitoana, Sporobolomyces roseus*
6 months	Laudis	2.4 × 10 ^a,A^	5.4 × 10 ^b,B^	*Alternaria, Arthrinium, Aspergillus, Fusarium, Penicillium griseofulvum, Ulocladium, Rhodotorula* spp., *Issatchenkia orientalis*
	Kangoo	9.7 × 10 ^a,A^	1.1 × 10 ^c,C^	*Aspergillus, Eurotium* (now *Aspergillus*)*, P. aurantiogriseum, P. citrinum, P. polonicum, Mycelia sterilia, Rhodotorula* spp., *Wickerhamomyces anomalus*
	Wintmalt	1.6 × 10 ^a,B^	8.6 × 10 ^b,A^	*Cladosporium, P. canescens, P. glabrum, P. polonicum, P. raistrickii, Mycelia sterilia, Candida saitoana, Sporobolomyces roseus*
9 months	Laudis	1.2 × 10 ^b,B^	3.5 × 10 ^c,C^	*Alternaria, Arthrinium, Cladosporium, Penicillium* spp., *Mycelia sterilia, Rhodotorula* spp., *Issatchenkia orientalis*
	Kangoo	7.9 × 10 ^c,A^	3.6 × 10 ^b,B^	*Alternaria, Arthrinium, Aspergillus, Cladosporium, Rhizopus, Rhodotorula* spp., *Wickerhamomyces anomalus*
	Wintmalt	1.3 × 10 ^a,B^	7.7 × 10 ^a,A^	*Aspergillus, Cladosporium, Rhizopus, Candida saitoana, Sporobolomyces roseus*
Floor warehouse				
3 months	Laudis	2.4 × 10 ^a,A^	1.0 × 10 ^c,D^	*Alternaria, Arthrinium, Cladosporium, Epicoccum, Fusarium, Giberella* (now *Fusarium*), *Rhodotorula* spp., *Issatchenkia orientalis*
	Kangoo	7.7 × 10 ^a,A^	<1 × 10	*Arthrinium, Aspergillus, Rhodotorula* spp., *Wickerhamomyces anomalus*
	Wintmalt	1.0 × 10 ^b,B^	2.3 × 10 ^b,B^	*Alternaria, Aspergillus, Mycelia sterilia, Penicillium aurantiogriseum, P. polonicum, P. griseofulvum, Candida saitoana, Sporobolomyces roseus*
6 months	Laudis	1.4 × 10 ^b,B^	1.8 × 10 ^b,D^	*Alternaria, Arthrinium, Cladosporium, Epicoccum, Mycelia sterilia, Rhodotorula* spp., *Issatchenkia orientalis*
	Kangoo	2.8 × 10 ^b,B^	-	*Rhodotorula* spp., *Wickerhamomyces anomalus*
	Wintmalt	1.1 × 10 ^b,B^	6.8 × 10 ^a,A^	*Alternaria, Cladosporium, Mycelia sterilia, Penicillium aurantiogriseum, P. polonicum, Candida saitoana, Sporobolomyces roseus*
9 months	Laudis	1.0 × 10 ^b,B^	2.2 × 10 ^a,D^	*Aspergillus, Penicillium brevicompactum, P. chrysogenum, Rhodotorula* spp., *Issatchenkia orientalis*
	Kangoo	2.2 × 10 ^a,B^	-	*Rhodotorula* spp., *Wickerhamomyces anomalus*
	Wintmalt	2.7 × 10 ^a,B^	-	*Candida saitoana, Sporobolomyces roseus*

Mean values with the same letters in columns were not significantly different (α = 0.05). The results for the indicated storage conditions and individual varieties are marked with small letters. The results for the varieties in the entire storage period, regardless of the conditions storage used, are marked with capital letters. Analyzing the data presented in Table 9, it is concluded that after 3 months of storage in silo, the count of the field fungi, respectively species of *Fusarium* and *Mucor,* decreased, and the development of stored grain specific fungi such as *Penicillium* and *Aspergillus* was observed. Altogether, 3 species of *Penicillium* were isolated from examined samples: 2 of them from the Wintmalt barley variety—*P. citrinum* and *P. griseofulvum*, and *P. aurantiogriseum* from the Kangoo variety. The quantitative representation of filamentous microscopic fungi was lower compared to yeasts, but higher compared to control grains, except the Wintmalt variety.

**Table 10:** Quantitative and qualitative composition of various groups of microorganisms in malt, depending on the time and type of storage of 3 varieties of barley.

Time of Storage	Varieties	Yeastlog CFU/g	Fungilog CFU/g	Species
0	Laudis	4.1 × 10 ^A^	1.8 × 10 ^C^	*Aspergillus, Cladosporium, Fusarium, Rhodotorula* spp., *Issatchenkia orientalis*
	Kangoo	2.2 × 10 ^A^	1.1 × 10 ^B^	*Aspergillus, Cladosporium, Mucor, Rhodotorula* spp., *Wickerhamomyces anomalus*
	Wintmalt	4.5 × 10 ^B^	2.2 × 10 ^C^	*Alternaria, Aspergillus, Cladosporium, Fusarium, Penicillium griseofulvum, Candida saitoana, Sporobolomyces roseus*
Silo				
3 months	Laudis	1.7 × 10 ^a,B^	1.6 × 10 ^b,C^	*Alternaria, Cladosporium, Penicillium corylophilum, Rhodotorula* spp., *Issatchenkia orientalis*
	Kangoo	2.3 × 10 ^a,A^	1.0 × 10 ^b,B^	*Alternaria, Aspergillus, Mucor, Rhodotorula* spp., *Wickerhamomyces anomalus*
	Wintmalt	1.8 × 10 ^a,C^	1.7 × 10 ^b,C^	*Alternaria, Penicillium griseofulvum, Ulocladium, Candida saitoana, Sporobolomyces roseus*
6 months	Laudis	2.0 × 10 ^a,B^	1.7 × 10 ^b,C^	*Alternaria, Cladosporium, Penicillium crustosum, Rhodotorula mucilaginosa, Rhodotorula* spp., *Issatchenkia orientalis*
	Kangoo	2.4 × 10 ^a,A^	1.3 × 10 ^b,B^	*Alternaria, Penicillium corylophilum, Rhodotorula* spp., *Wickerhamomyces anomalus*
	Wintmalt	1.8 × 10 ^a,C^	2.2 × 10 ^b,C^	*Alternaria, Mucor, Candida saitoana, Sporobolomyces roseus*
9 months	Laudis	1.6 × 10 ^a,B^	3.3 × 10 ^a,B^	*Alternaria, Cladosporium, Mucor*, *Mycelia sterilia, Rhodotorula mucilaginosa, Rhodotorula* spp., *Issatchenkia orientalis*
	Kangoo	1.4 × 10 ^b,AB^	2.2 × 10 ^a,B^	*Alternaria, Mucor, Rhodotorula* spp., *Wickerhamomyces anomalus*
	Wintmalt	1.3 × 10 ^a,C^	5.4 × 10 ^a,B^	*Alternaria, Cladosporium, Candida saitoana, Sporobolomyces roseus*
Floor warehouse				
3 months	Laudis	2.1 × 10 ^a,B^	1.8 × 10 ^b,C^	*Alternaria, Epicoccum, Penicillium corylophilum, Rhodotorula* spp., *Issatchenkia orientalis*
	Kangoo	1.7 × 10 ^ab,AB^	1.1 × 10 ^b,B^	*Alternaria, Absidia, Rhodotorula* spp., *Wickerhamomyces anomalus*
	Wintmalt	6.8 × 10 ^a,A^	1.9 × 10 ^b,C^	*Alternaria, Cladosporium, Candida saitoana, Sporobolomyces roseus*
6 months	Laudis	1.8 × 10 ^a,B^	2.7 × 10 ^b,B^	*Alternaria, Mucor, P. aurantiogriseum, Rhodotorula mucilaginosa, Rhodotorula* spp., *Issatchenkia orientalis*
	Kangoo	2.5 × 10 ^a,A^	8.1 × 10 ^a,A^	*Alternaria, Mucor, Rhodotorula* spp., *Wickerhamomyces anomalus*
	Wintmalt	2.2 × 10 ^b,BC^	7.7 × 10 ^a,A^	*Penicillium griseofulvum, Rhizopus, Candida saitoana, Sporobolomyces roseus*
9 months	Laudis	1.0 × 10 ^b,B^	8.3 × 10 ^a,A^	*Penicillium crustosum, Rhodotorula mucilaginosa*
	Kangoo	1.0 × 10 ^b,B^	1.8 × 10 ^b,B^	*Mucor, Rhizopus, Rhodotorula* spp., *Wickerhamomyces anomalus*
	Wintmalt	3.1 × 10 ^b,B^	1.3 × 10 ^b,C^	*P. chrysogenum, P.* spp., *Candida saitoana, Sporobolomyces roseus*

Mean values with the same letters in columns were not significantly different (α = 0.05). The results for the indicated storage conditions and individual varieties are marked with small letters. The results for the varieties in the entire storage period, regardless of the conditions of storage used, are marked with capital letters.

**Table 11:** Toxigenicity of selected *Penicillium* strains, isolated from barley and malt.

Species	P	G	RC	CPA	PA
Toxigenicity from barley by the plate dilution method					
*P. griseofulvum*	5 */6 **	6/6	0/6	6/6	
*P. chrysogenum*			3/3		
*P. raistrickii*		5/5			
Toxigenicity from malt by the plate dilution method					
*P. chrysogenum*			0/2		
Toxigenicity from endogenous mycobiota of barley					
*P. crustosum*			0/2		2/2
*P. hordei*			1/1		
*P. expansum*	3/3	3/3	0/3		
*P. griseofulvum*	6/8	7/8	0/8	7/8	
*P. chrysogenum*			0/3		
*P. raistrickii*		8/8			
Toxigenicity from endogenous mycobiota of malt					
*P. griseofulvum*	1/2	2/2	0/2	2/2	
*P. chrysogenum*			0/1		
*P. raistrickii*		3/3			

*—number of isolates with ability to produce mycotoxins, **—number of tested isolates, P—patulin, G—griseofulvin, RC—roquefortin C, CPA—cyclopiazonic acid, PA—penitrem A.

## Data Availability

The datasets generated during and/or analyzed during the current study are available from the corresponding author on reasonable request.
